# Supplementary material for: Empirical methods for controlling false positives and estimating confidence in ChIP-Seq peaks
Source: BMC Bioinformatics. 2008 Dec 5;9:523. doi: 10.1186/1471-2105-9-523 (PMC2628906; doi:10.1186/1471-2105-9-523)
Supplement: Additional File 1 — A variety of html documents from the USeq web site detailing the available applications, their best usage, output file type descriptions, command line menus, etc. [file 1471-2105-9-523-S1.zip › USeqUserGuides/JavaDoc.zip!/JavaDoc/bioroot/oligo/class-use/OligoBean.html]

Uses of Class bioroot.oligo.OligoBean


|  |  |  |
| --- | --- | --- |
| **Overview** \| **Package** \| **Class** \| **Use** \| **Tree** \| **Deprecated** \| **Index** \| **Help** | |  |
| PREV   NEXT | **FRAMES**    **NO FRAMES**     **All Classes** |  |


---


## **Uses of Class bioroot.oligo.OligoBean**

| Packages that use OligoBean | |
| --- | --- |
| **bioroot** |  |
| **bioroot.oligo** |  |

Uses of OligoBean in bioroot

| Methods in bioroot that return OligoBean | |
| --- | --- |
| `static OligoBean[]` | `Archive.makeOligoBeans(DBUtil bioRoot, UserBean userBean)`             Makes an array of all visibile OligoBeans |

Uses of OligoBean in bioroot.oligo

| Methods in bioroot.oligo that return OligoBean | |
| --- | --- |
| `static OligoBean[]` | `OligoSpreadSheet.fetchOligoBeans(java.lang.String sql, DBUtil bioRoot)`             Build OligoBean[] from Oligo table in database. |
| `OligoBean[]` | `OligoUploadBean.getOligoBeans()` |
| `static OligoBean[]` | `OligoSpreadSheet.orderOligos(java.lang.String featureName, Preference pref, OligoBean[] beans, DBUtil bioRoot)`             Orders oligos, sort of nasty, can't figure another way, reflection is way too slow. |
| `static OligoBean[]` | `OligoBase.reverseOligoBeans(OligoBean[] beans)`             Reverses the order of an array of OligoBean. |

| Methods in bioroot.oligo with parameters of type OligoBean | |
| --- | --- |
| `static java.lang.String` | `OligoModify.deleteOligo(OligoBean oligoBean, UserBean userBean, DBUtil bioRoot)`             Attempts to delete an oligo, if sucessful, returns a "" String, otherwise an error message. |
| `static java.lang.String` | `OligoBase.fetchConcentrationGroup(OligoBean ob)`             Returns a concentration unit |
| `static java.lang.String` | `OligoSpreadSheet.fetchOligoTableRows(Preference pref, OligoBean[] beans, int[] startStop, DBUtil bioRoot, boolean checked, boolean allTabs)`             Returns table rows for every oligo according to an OligoPreference, if allTabs==true, then all features are returned in a tab delimited format, otherwise its HTML. |
| `static java.lang.String` | `OligoBase.getOligoForm(OligoBean ob, DBUtil bioRoot, UserBean userBean)`             Returns an oligo form filled it with the values in the oligo bean. |
| `static java.lang.String` | `OligoBase.getOligoReport(OligoBean ob, DBUtil bioRoot, UserBean userBean, boolean commonForm)`             Returns an oligo report. |
| `static OligoBean[]` | `OligoSpreadSheet.orderOligos(java.lang.String featureName, Preference pref, OligoBean[] beans, DBUtil bioRoot)`             Orders oligos, sort of nasty, can't figure another way, reflection is way too slow. |
| `static OligoBean[]` | `OligoBase.reverseOligoBeans(OligoBean[] beans)`             Reverses the order of an array of OligoBean. |
| `void` | `OligoUploadBean.setOligoBeans(OligoBean[] beans)` |
| `static boolean` | `OligoModify.subtractAndSetOligoBeans(OligoBean[] oligoBeans, javax.servlet.http.HttpSession session)`             Subtracts a bean from the OligoBean[] and resets it in oligoBeansToEdit |
| `static java.lang.String` | `OligoModify.updateOligo(OligoBean oligoBean, UserBean userBean, javax.servlet.http.HttpServletRequest request, DBUtil bioRoot)`             Attempts to update an OligoBean in the database, only checks that the name is unique if it has changed. |

---


|  |  |  |
| --- | --- | --- |
| **Overview** \| **Package** \| **Class** \| **Use** \| **Tree** \| **Deprecated** \| **Index** \| **Help** | |  |
| PREV   NEXT | **FRAMES**    **NO FRAMES**     **All Classes** |  |


---
